# Case Report of Dysmorphic Physical Findings in Male With Shapiro Syndrome

**DOI:** 10.7759/cureus.26064

**Published:** 2022-06-18

**Authors:** Catherine M Evers Smith, Eric Creed

**Affiliations:** 1 Neurology, Wright State University Boonshoft School of Medicine, Dayton, USA; 2 Neurology, University of North Carolina School of Medicine, Chapel Hill, USA

**Keywords:** dysmorphic features, episodic hypothermia, hyperhidrosis, agenesis of the corpus callosum, shapiro syndrome

## Abstract

Shapiro syndrome is an extremely rare disorder characterized by a triad of episodic hypothermia below 95 °F (35°C), hyperhidrosis, and agenesis/dysgenesis of the corpus callosum. The exact mechanism is unknown. Based on a review of the literature, this is the first reported case of dysmorphic physical exam findings in Shapiro syndrome. This case suggests the possibility of an underlying genetic disorder in Shapiro syndrome.

## Introduction

Shapiro syndrome (spontaneous recurrent hypothermia with corpus callosum dysgenesis), first described in 1969 by Shapiro, is a rare disorder consisting of the triad of paroxysmal episodic hypothermia, hyperhidrosis, and agenesis/dysgenesis of the corpus callosum [1]. Hypothermia below 95 °F (35°C) and hyperhidrosis have been found to be the most prevalent symptoms [2]. The mechanism is not fully understood. The presence of the same syndrome in siblings may be due to a genetic component or an environmental exposure component [2]. There is no definitive treatment. Treatments are aimed at increasing patient body temperature during hypothermic episodes and include external warming, such as warming blankets or systems, as well as centrally-acting medications, such as clonidine [2-3]. Our case is the first reported case of dysmorphic physical exam findings in Shapiro syndrome. This case suggests the possibility of an underlying genetic disorder in Shapiro syndrome or an association with other syndromes and highlights the importance of a thorough physical exam in all patients. 

## Case presentation

**Figure 1:**
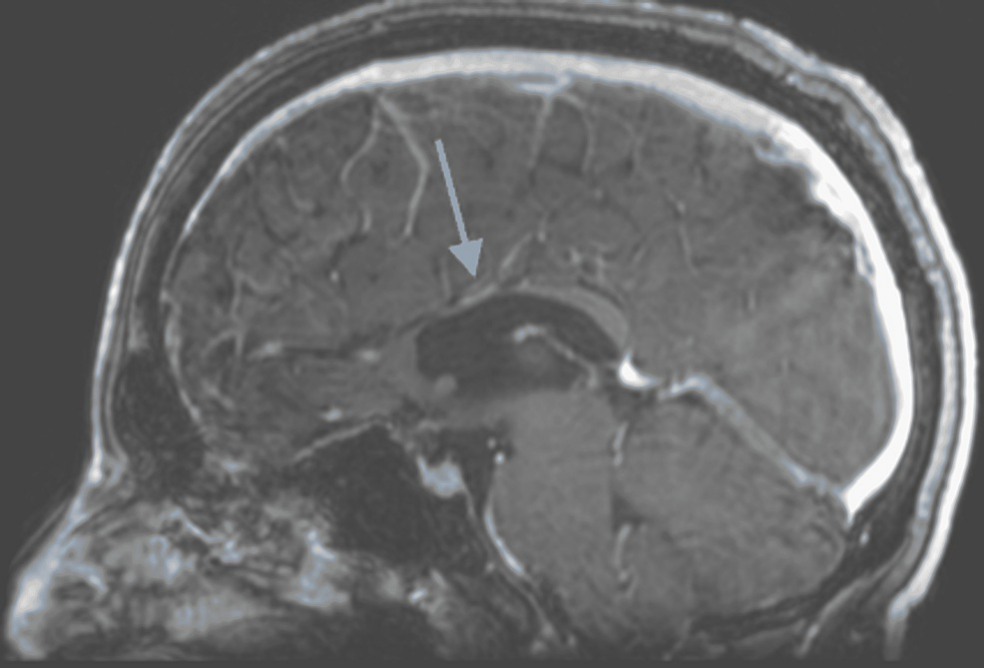
MRI head with an arrow identifying corpus callosum agenesis

**Figure 2:**
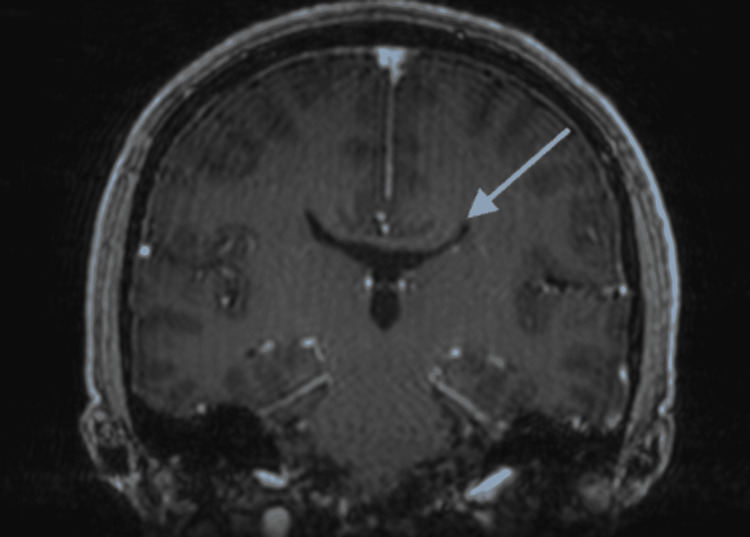
MRI head demonstrating Viking's helmet appearance of the lateral ventricles

The patient is a 35-year-old male who presented with a chief complaint of temperature fluctuations dating back to November 2020. Episodes alternated between severe diffuse diaphoresis and hypothermia with chills, occurred two to three times per day, and lasted a few hours before abating. On initial arrival, the oral temperature was 92° F (33.3°C). Initial physical examination revealed child-like facies, wide and flat nasal bridge, hypotelorism, geographic tongue, and diffuse diaphoresis. Magnetic resonance imaging (MRI) of the brain demonstrated dysgenesis of the corpus callosum (Figure 1) and Viking's helmet appearance of the lateral ventricles, which is seen in conditions with corpus callosum agenesis (Figure 2).

**Figure 3:**
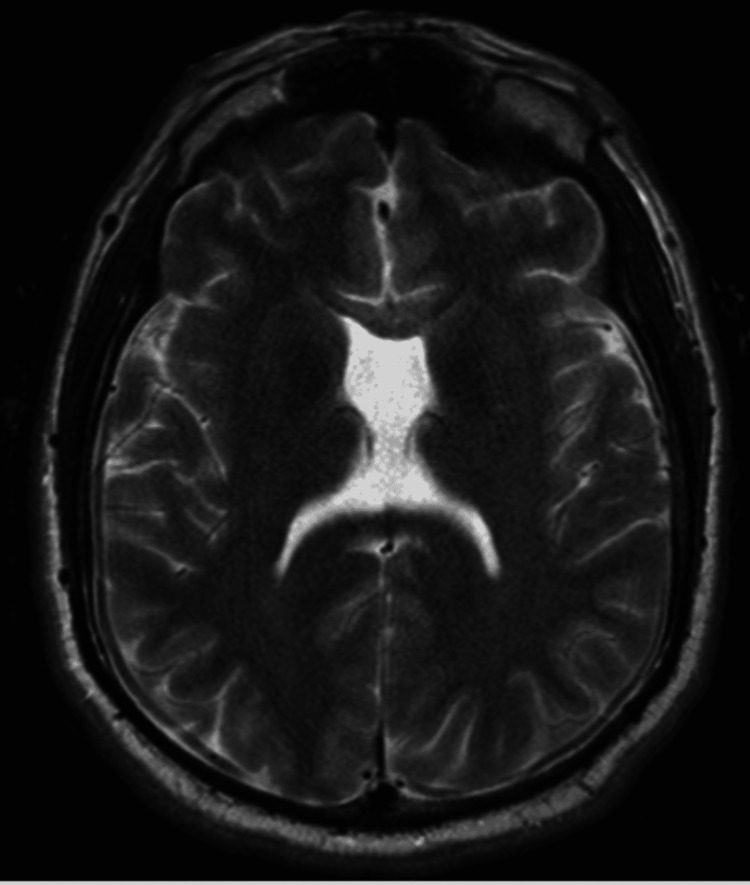
MRI demonstrating absent nasal septum possibly secondary to holoprosencephaly

The patient was subsequently transferred to an outside hospital where an MRI pituitary was obtained (Figure 3), demonstrating no pituitary pathologies on imaging but re-demonstrating severe corpus callosum dysgenesis as well as absent septum pellucidum with hypoplastic cingulate gyrus, possibly fused anteriorly, and azygos configuration of the anterior cerebral artery, possibly representing mild holoprosencephaly or lobar holoprosencephaly.

During outside admission, the patient also had episodes of bradycardia as low as 39 beats per minute, requiring atropine as well as hypothermia as low as 93.4° F (34.1°C), requiring intermittent Bair hugger™ (3M, Saint Paul, Minnesota). The differential diagnosis for hypothermia includes disorders causing increased heat loss, impaired temperature regulation, or decreased heat production. During his admissions, the patient underwent extensive laboratory evaluation, including complete blood count with differential, parathyroid hormone, thyroid-stimulating hormone, urinary analysis, procalcitonin, quantiferon testing, blood cultures, Covid testing, lipase, liver function tests, urine drug screen, screens for various infectious etiologies (multiple viral and bacterial species), and cerebrospinal fluid analysis, all of which were within normal limits. Given the patient's fluctuating symptoms of hypothermia and diaphoresis, combined with findings on intracranial imaging of agenesis/dysgenesis of the corpus callosum, the most likely diagnosis was deduced to be Shapiro syndrome. Genetic studies were not performed at that time per patient preference. 

## Discussion

Shapiro syndrome is an extremely rare disorder that was first described by Shapiro in 1969 as a triad of paroxysmal episodic hypothermia, hyperhidrosis, and agenesis of the corpus callosum [1]. A review paper from 2014 recorded a total of 52 cases from an extensive literature review from 1934 to 2013 [2]. The typical presentation includes corpus callosum agenesis, which is reported in only 40% of all cases. There is also a variant form that refers to Shapiro syndrome in the absence of corpus callosum. Episodic hypothermia is considered the defining hallmark as it is reported in both the typical and the variant forms of Shapiro Syndrome. Hyperhidrosis, which is also considered part of the triad hallmarking Shapiro syndrome, was found to be present only in 42.3% of all cases [2]. Hyperhidrosis was found mainly in adult-onset cases. Other common symptoms include pallor, altered consciousness, chills, and bradycardia (with a prior report of ventricular fibrillation requiring a pacemaker) [2-3].

The central regulation of body temperature involves multiple areas of the brain, including the hypothalamus, limbic system, brain stem, spinal cord, and sympathetic ganglia [2,4]. The hypothalamus is the thermoregulation control center, with heat dissipation and heat conservation being controlled by the anterior and poster centers, respectively [5]. The mechanism of periodic hypothermia with agenesis of the corpus callosum is not fully understood. Postulated mechanisms include degenerative or inflammatory processes leading to an epileptic focus explaining the periodic dysfunction [4]. 

Treatment of this disorder involves warming the patient during episodes of hypothermia. Warming blankets and systems can be used as well as a variety of medications. There is no definitive treatment for the disorder, so most medications are centrally acting and aimed at mimicking neurotransmitters affecting the hypothalamus [6]. Clonidine is the most commonly used. Other options include gabapentin, venlafaxine, chlorpromazine, and levodopa-carbidopa [6]. These can be used individually or as combinations. A minority of patients are refractory to treatment [2]. 

A genetic component to Shapiro syndrome has been suggested based on reports of Shapiro syndrome in siblings [2]. These reports include siblings of different genders, suggesting an autosomal mutation, possible recessive or de novo due to gonadic mosaicism [2]. Our case is the first to report dysmorphic findings in Shapiro syndrome. There was a previously documented case report of holoprosencephaly with Shapiro syndrome [7]. This patient’s hypotelorism could be secondary to holoprosencephaly, which is supported by findings on MRI brain consistent with holoprosencephaly.

## Conclusions

It has been postulated that Shapiro syndrome is a genetic syndrome, possibly autosomal recessive (given the history of siblings with the condition). Our case findings with dysmorphic features, which have not previously been reported in patients with Shapiro syndrome, further point towards an underlying genetic cause. This case further demonstrates the importance of a complete physical examination in neurological patients.
